# The Native Speaker in Italian-Dialects Bilingualism: Insights From the Acquisition of Vicentino by Preschool Children

**DOI:** 10.3389/fpsyg.2021.717639

**Published:** 2021-10-15

**Authors:** Emanuela Sanfelici, Maja Roch

**Affiliations:** ^1^Department of Linguistic and Literary Studies, University of Padua, Padua, Italy; ^2^Department of Psychology, University of Padua, Padua, Italy

**Keywords:** bilingualism, Italian dialect, comprehension, production, preschool children

## Abstract

This paper investigates the bilingualism originating from the native competence of a standard language (Italian) and a vernacular non-standardized local dialect (henceforth, bilectalism). We report results on the comprehension and production of narrative stories by 44 3- to 5-year-old typically developing children exposed to both Italian and Vicentino from birth. Our findings show that all children produced and comprehended Italian. As for the dialect, children can comprehend Vicentino, despite not producing any dialectal element. The study further revealed an implicational scale in dialectal competence: if a child exhibits some productions with dialectal syntax, s/he also produces dialects at the phonological, morphological, and lexical levels. These findings are in line with the dialectological studies on adult speakers: dialectal competence should be arranged along a fine-grained continuum and the dialectal speaker should be considered as a multi-factorial notion. Our study extends this observation to children’s dialectal acquisition.

## Introduction

Recent linguistic and psychological literature has questioned the standard definition of the native speaker suggesting that nativeness is an articulated, composite, and dynamic state which may result from the interaction between linguistic cognition and extra-linguistic factors, e.g., literacy (Davies, 2003, 2013; Sorace, 2003; Hulstijn, 2015; a.m.o.). Whereas much attention has been paid to second language acquisition, bilingualism in two majority language contexts, and heritage language acquisition, little is known about the notion of nativeness and, more generally, about the acquisition modes in bilectal contexts (Lauchlan et al., 2012; Vangsnes et al., 2015; Antoniou et al., 2016; Leivada et al., 2017) and even less about the acquisition of Italian-dialects bilectalism (with the exception of Sardinian, Garraffa et al., 2015, 2017; Klaschik and Kupisch, 2016; Kupisch and Klaschik, 2017).

Italian-dialects bilectalism provides an ideal ground for a deeper understanding of nativeness. Indeed, sociolinguistic and dialectological studies on Italian dialects have demonstrated that, besides sharing some features with other types of bilingualism, Italian bilectalism exhibits at least three outstanding properties. First, whereas the whole community usually comprehends both Italian and the local dialect and speaks Italian, only a portion of the community can also speak the local dialect (Mioni and Trumper, 1977; Mioni, 1979, 2007; Marcato and Ursini, 1998; Loporcaro, 2013; Garraffa et al., 2017; a.o.). The ISTAT (2015) survey revealed that more than half of the Italian population from age six can only speak Italian (55.1%). Both dialect and Italian are used in everyday life by 25.2% of Italians, while only 12.2% actively speak the local dialect.^1^ The low proportion of dialectal speakers may affect the quantity of dialectal input children are exposed to. Children may be more frequently exposed to Italian since Italian is likely to be used more frequently, e.g., in school and the media, and by many different native speakers. Second, the local dialects and Italian are used in different sociolinguistic domains (Mioni, 1979, 2007; Berruto, 2006; a.m.o.). Dialects are often bound to specific and limited contexts, like home language. While the majority of Italians speak exclusively Italian when interacting with strangers (79.5%) and friends (49.6%), Italians speak both languages (32%) or only dialect (14.1%) at home, according to the ISTAT (2015) survey. Since hearing a language from several different speakers is more supportive of language development than hearing a language for the same number of hours from fewer speakers (Houston and Jusczyk, 2000; Hoff, 2015), the lack of multiple speakers may lead to a less rich and less varied dialectal input. Third, dialects often lack a written standard: with very few exceptions (e.g., Friulian), no children’s books and no media activities are available in dialect. In addition, minority languages and dialects usually lack official recognition, which implies less or null support at the educational level. It is usually the case that the local dialect is not taught in school. Since all these extra-linguistic properties plausibly influence input consistency, bilectal acquisition may differ from the acquisition in standard bilingual contexts.

The dialectological literature has also demonstrated that dialectal competence is arranged along a fine-grained continuum and that dialectal speakers do not have a fixed position on this scale along their life span (Mioni, 1979; Berruto, 2005, 2006; Benincà and Poletto, 2006; Cerruti, 2011; a.o.). Various studies on adults and school-aged speakers have shown that, while only a few students attending middle schools declared they were competent in their local dialect, the number of dialectal speakers increased with age (Berruto, 2005; Marcato, 2005; Berruto, 2007; Tessarolo and Bordon, 2015). While adults’ and adolescents’ dialectal competence has been quite extensively investigated, little information is available on the early stages of dialectal acquisition (Cardinaletti, 2013; Bonifacio, 2014; Garraffa et al., 2015; Klaschik and Kupisch, 2016; Kupisch and Klaschik, 2017; Covazzi, 2019). Some studies have investigated children’s dialectal production (Cardinaletti, 2013; Bonifacio, 2014; Klaschik and Kupisch, 2016; Kupisch and Klaschik, 2017; Covazzi, 2019), whereas others have tested comprehension (Garraffa et al., 2015). All these acquisition studies have demonstrated that, although Italian is the dominant language, at least some children are competent in the local dialect. Children’s dialectal competence varies across children and across the linguistic phenomena investigated in the studies. Furthermore, according to some scholars, children’s dialectal production depends on the quantity of dialectal input children have been exposed to Klaschik and Kupisch (2016), Kupisch and Klaschik (2017); but see Covazzi (2019). We lack a study that assesses both comprehension, production, and quantity of input in bilectal children.

Building on the previous acquisition studies, we investigate both production and comprehension of Vicentino, a Venetan dialect, by preschool children from age 3 to 5. Through a parental questionnaire, we measure the quantity of dialectal input to verify to which extent children’s production and comprehension correlate with language exposure. The broad aim of our study is to assess the nature and the status of the bilectal preschool speaker, by asking whether preschool children comprehend and produce any dialect.

The structure of the paper is as follows. In Section “Previous studies on early bilectal acquisition” we summarize previous studies on early bilectal acquisition. In Section “The Vicentino dialect” we outline some notes on the sociolinguistic status of Vicentino and delineate those grammatical properties differentiating Vicentino from Italian, that become relevant in the analysis of children’s productions. Section “Current study” presents the study, while Section “Results” reports the results. Finally, in Section “Discussion” we discuss our findings and conclude the paper.

## Previous Studies on Early Bilectal Acquisition

In the last decade, scholars have started investigating early bilectal acquisition, asking how bilectal children perform in grammatical and cognitive tasks and how they acquire various dialectal grammatical properties. Some studies have demonstrated that bilectal children’s performance is not distinct from that of monolingual children, and, when differences emerge, bilectal children outperform monolinguals (Cardinaletti, 2013; Garraffa et al., 2015). Garraffa et al. (2015) investigated receptive Italian grammatical competence and general cognitive abilities in bilectal Italian-Sardinian children attending primary school. Their results showed that the performance of Sardinian–Italian bilectal children was generally indistinguishable from that of monolingual Italian children, in terms of both Italian language skills and cognitive abilities. Where differences were detected, these were mostly in favor of bilectal children. A similar conclusion was reached by Cardinaletti (2013), who analyzed the emergence of clitics in the spontaneous productions of one bilectal Italian–Venetan child aged 2–3 years, one monolingual Venetan child, and one age-matched Italian monolingual child. In the bilectal child, object clitics emerged roughly at the same time in Italian and Venetan, and omissions stopped at the same time in both languages. Conversely, in the two monolingual children object clitics were omitted at a much higher rate and for a longer period than in the bilectal child. The author concluded that converging evidence from two close languages, Italian and Venetan, speeds up the acquisition of object clitics. Conversely, Bonifacio (2014) did not find a similar advantage. The author investigated the acquisition of the copula in the spontaneous productions of two bilectal Italian-Venetan children (Age 19–27 months and 24–36 months) and one Italian monolingual child aged 22–28 months. Although the three children exhibited a very similar developmental path in copula omission and production as well as the agreement between the copula and the subject, they differed in the productions of articles in copular contexts. While the Italian monolingual child produced articles in a target-like fashion, the two bilectal children were not fully competent, producing very few articles.

Only three studies we are aware of provide information concerning the quantity of dialectal input children are exposed to and children’s dialectal productions (Klaschik and Kupisch, 2016; Kupisch and Klaschik, 2017; Covazzi, 2019). Klaschik and Kupisch (2016) tested the interpretation of subjects in Italian and Venetian by 20 Italian–Venetian children aged 7–12 years. Bilectal speakers were reported to accept more overt subjects when the discourse topic was maintained compared to monolingual Italian speakers. This result suggests that there was an influence from Venetian, where overt subjects are mandatory in some contexts. In addition, those children who were reported to use dialect in everyday life comparatively more often outperformed children who were reported to be infrequent dialect users. The authors concluded that exposure to a dialect does not negatively affect the use of the standard language. Kupisch and Klaschik (2017) study dialectal influence and gender marking in 25 bilectal Italian–Venetan children aged 5–11 years. Participants were tested with an elicited production task in both Italian and Venetan. Children followed the gender assignment rules of Italian and Venetan. Interestingly, no dialectal influence was detected in the Italian experiment: children produced only Italian DPs. Conversely, in the Venetan experiment, the production was much more varied: children produced Italian, Venetan, and mixed DPs. The degree of dialectal use was suggested to be dependent on the quantity of dialectal input children may have been exposed to. A slightly different conclusion was reached by Covazzi (2019), investigating the production of relative clauses by 23 preschool bilingual Italian–Friulian children aged 4–6 years. In line with much cross-linguistic research, she found that the production of subject relative clauses was more accurate than that of object relatives. Although Italian was the dominant language and children’s production was essentially Italian, the author reported that specific influences of Friulian on Italian were indeed present in children’s productions. However, no correlation was found between children’s Friulian productions and the quantity of Friulian input children were reported to receive in the parental questionnaire.

Taking the findings together, we conclude that, although children may be competent in the dialect, the degree of children’s dialectal production and comprehension seems to depend on the linguistic phenomenon investigated and the quantity of dialectal input. Our study adds to these previous acquisition works by testing both comprehension and production and by including a measure of dialectal input. Before illustrating the study, we outline some notes on the sociolinguistic status and grammatical properties of Vicentino.

## The Vicentino Dialect

Venetan is a Western Romance language. Similar to other varieties, Venetan has developed independently from Latin. Thus, it is not a dialect of Italian from a genealogical perspective. As Zamboni (1974, 1979) proposed, Venetan comprises different language groups: *the Venetian group* (Venezia), *the central group* (Padova, Vicenza, Polesine), *the North Venetan group* (also labeled trevigiano-feltrino-bellunese), the *outer Venetan* spoken in the Trento province, and the varieties spoken in Friuli Venezia-Giulia, Istria and Dalmazia (Zamboni, 1974, 1989; Cortelazzo, 1981; Loporcaro, 2013).

Vicentino is a Venetan dialect, spoken in the Vicenza province located in the Veneto region (Pellegrini, 1977). In 2007, the Veneto regional administration issued a law (L.R. 13.4.2007, n. 8) promoting and financially supporting initiatives to study and preserve the use of the local Venetan dialects. Thanks to this law and the regional economic growth between the ’80s and ’90s, Venetan dialects, including Vicentino, have recently experienced an increase in their prestige, especially among young people aged 25–34 years (Santipolo and Tucciarone, 2006). According to the ISTAT (2015) survey carried out more than half of the Veneto population speak the local dialects at home. One third of the Veneto population exclusively speak a Venetan dialect at home (30.6%), one third use both Italian and a Venetan dialect (31.4%), while the remaining Veneto population exclusively use Italian (28.5%). A similar observation holds for the use of dialect when speakers interact with friends: 28.7% of the population exclusively use a Venetan dialect, 33.6% use both Italian and a Venetan dialect, while 30.6% only speak Italian. With strangers, the use of dialect decreases: people speak either exclusively Italian (65.6%) or use both Italian and dialect (23.1%), whereas only 8.7% only speak the local dialect.

Italian and Vicentino differ with respect to several phonological, morphological, syntactic, and lexical properties. In this section, we provide a summary of the properties differentiating Vicentino from Italian: the list is by no means exhaustive but contains only those features relevant to the results in Section “Results.”

Some grammatical features of Vicentino are shared by other Venetan dialects, whereas others are peculiar to this group. Unless otherwise specified, we do not distinguish when the features are common to the Venetan dialects or only specific to the central Venetan ones, to the extent that they differ from those of Italian.

Among the phonological dialectal features, we find voicing of degeminated plosive in intervocalic contexts: degeminated voiceless plosive consonants become voiced in intervocalic contexts, [rɔ:da] for *ruota* ‘wheel,’ [manè:go] for *manico* ‘handle’ [see ex. (6) in Section “Results”]. In addition, phonological double plosives undergo degemination in intervocalic contexts: [katì:vo] instead of the Italian [kattì:vo] ‘bad’ (Zamboni, 1974; Loporcaro, 2013, p. 86). Likewise, the lateral consonant/l/in intervocalic contexts undergoes degemination when geminated, [bà:la] instead of the Italian [pàlla] ‘ball’ (Zamboni, 1974, p. 37) [see ex. (6) in Section “Results”]. Vicentino also has the apocope of the final unstressed vowels/e/and/o/. In other Veneto dialects, i.e., Venetian, apocope of the final unstressed vowel/e/occurs after degeminated/n, l, r/, while/o/is dropped only when following/n/: [kà:ŋ], [sa:l], [saòr] instead of the Italian [kà:ne] ‘dog,’ [sà:le] ‘salt’ and [sapò:re] ‘taste’; [feŋ], [pjeŋ] instead of [fjeno] ‘hay’ and [pjeno] ‘full.’ In Vicentino, apocope of/e,o/is more restricted: it can only occur when following/n/(see Loporcaro, 2013, p. 86, 105). Hence, while in Vicentino ‘dog’ is [kà:ŋ], the word for ‘salt’ is [sà:le], without the apocope of/e/. Notice that the final/n/undergoes velarization after apocope [see ex. (4–5) in Section “Results”].

Among the morphological features, we quote the unique form of the masculine singular definite article which is a weak form and is *el*, instead of the *lo/il* of Italian (Marcato and Ursini, 1998, p. 86).

A pervasive feature of the Vicentino dialect is the productive use of prefixes attached to denominal and deadjectival verbs to encode various aspectual values (Marcato and Ursini, 1998, p. 230). A case in point is represented by the prefix *in-*: *in-rabiarse* (It. *arrabbiarsi*) ‘to get angry,’ *in-tardigare* (It. *tardare*) ‘to be late,’ *in-marsire* (It. *marcire*) ‘to rot.’ Another instance is the use of the prefix *s-* instead of the Italian *in*-, as in *s-cumissiare* (It. *in-cominciare*) ‘to begin, start.’

As for the morpho-syntax, Vicentino productively uses particle verbs. Some examples are *dire sù* ‘lit. to speak up; to reproach,’ *saltare sù* ‘lit. to jump up; to attack,’ *torre sù* ‘lit. to take up; to pick up,’ *molare zò* ‘lit. to release down; to release.’

While in Italian the progressive imperfective aspect is encoded by the construction *stare* ‘to stay’ plus gerund, Vicentino uses the construction *essere/essare drio* ‘lit. to be behind’ plus bare infinitive as exemplified in (1) [see ex. (6) in Section ‘‘Results’’].^2^


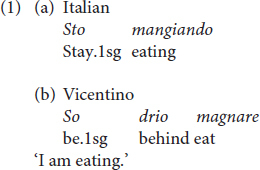


Another difference between Italian and Vicentino lies in the pro-drop parameter value. As is well-known, Italian is a pro-drop language (see Rizzi, 1982). Conversely, Vicentino does not generally allow a pro subject: subject clitics for III singular and plural person must be lexicalized when the predicate is finite (see Benincà and Vanelli, 1982, p. 41; Marcato and Ursini, 1998; we refer the reader to Benincà, 1994; Poletto, 1996 for a more precise picture on subject clitics in Vicentino). With existential predicates, Vicentino exhibits the locative clitic *ghe* (Benincà and Vanelli, 1982), as in *ghe jera un can* ‘there was a dog.’

Another difference between Italian and Vicentino syntax regards auxiliary selection. In finite contexts, in Vicentino causative transitive, impersonal, antipassive, and reflexive predicates allow both the auxiliary *ésar* ‘to be’ and *gavere* ‘to have,’ with a general preference for *gavere* (Gagarina et al., 2012; Benincà, 1994; a.o.). Conversely, in Italian, these predicates only select the auxiliary ‘to be.’ Compare the Vicentino examples in (a) with the Italian counterpart in (b) (see ex. (7) in Section “Results”).


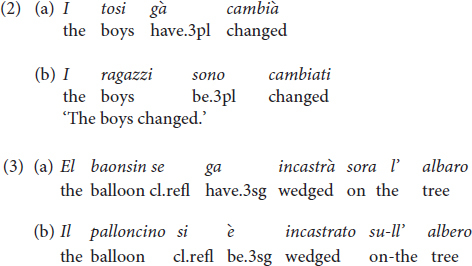


Finally, we quote the presence of the doubly filled comp in various subordinating conjunctions, which is absent in Italian: the complementizer *che* follows the wh-phrase *come* ‘how,’ i.e. *come che* ‘how,’ and the adverbial *péna* ‘soon,’ i.e. *péna che* ‘as soon as’ (Marcato and Ursini, 1998). We further notice that Vicentino temporal subordinate clauses are introduced by the lexical item *có* instead of the corresponding Italian *quando* ‘when’ (see ex. (8) in Section “Results”).

Although this linguistic profile is not exhaustive, it allows us to detect clear phenomena in which the Vicentino dialect and Italian diverge and thus to characterize the linguistic productions by establishing the type and the degree of dialectal features children produce.

## Current Study

We investigated the comprehension and the production of narrative stories in bilectal preschool children who have been exposed to Italian and Vicentino from birth. Participants were tested with the picture-supported task “Multilingual Assessment Instrument for Narratives” (MAIN, Gagarina et al., 2012, 2015; Gagarina and Lindgren, 2020) in two experimental sessions, one in Italian and the other in Vicentino. In addition, parental questionnaires were collected to obtain information concerning the family socioeconomic status as well as children’s language exposure.

Since no studies we are aware of have tested both comprehension and production in this bilectal context, our broad research question was to determine preschool children’s dialectal competence in both production and comprehension. Four research questions were formulated.

(Q1) Does children’s performance in narrative comprehension and production differ depending on the mode of the narrative task, i.e., retelling vs. telling?

We expect a difference in the narrative mode. We hypothesize the complexity, coherence, and accuracy of narrative stories, i.e., macrostructure, to increase when the child is presented first with a well-structured and coherent model story and then asked to retell the story. This hypothesis is based on various studies on bilinguals showing that children comprehended better and produced more structured and coherent stories with the retelling task than with the telling task (e.g., Kunnari et al., 2016; Maviş et al., 2016; Levorato and Roch, 2020).

(Q2) Are there differences in narrative abilities between children determined by age?

A difference in age is also hypothesized given previous studies which demonstrated that children’s narrative abilities increase with age (e.g., Pearson, 2002; Florit et al., 2014). This should hold for the complexity, coherence, and accuracy of children’s narratives (Bohnacker, 2016; Bonifacci et al., 2018).

(Q3) Does children’s performance in narrative comprehension and production differ depending on the language of the experiment, i.e., Vicentino vs. Italian?

Question (3) forms the bulk of this study. If bilectalism is similar to the bilingualism found in other contexts, we expect to find no differences depending on the language of administration, especially in the macrostructure. Story macrostructure is reported to be less dependent on language abilities as compared to microstructure, as typically operationalized in number of sentences and words (Berman and Slobin, 1994; Rodina, 2016). In addition, various studies have proposed that there might be a carry-over of the particular macrostructure elements across the bilingual’s two languages, even if the child’s linguistic abilities in one of them are weaker (Gagarina, 2016). As in previous bilingual studies, if there is a difference depending on the language, we expect this to affect the microstructure. Given previous dialectological and sociolinguistic findings reported in Sections “Introduction” and “Previous studies on early bilectal acquisition,” we hypothesize the acquisition of dialect to lag behind that of Italian.

(Q4) Does children’s competence depend on the quantity of input?

We expect the quantity of input to affect children’s performance. To date, studies have indicated that the quantity of language exposure is a crucial factor in language development. The quantity of language exposure has been reported to significantly influence the size of children’s vocabularies (Hoff et al., 2014) as well as the morpho-syntactic development and language proficiency (Hoff, 2015; Dicataldo et al., 2020). Since microstructure measures of bilingual narratives also reflect the lexical and morpho-syntactic development of each of their languages, we hypothesize that we will detect the effect more in the microstructure than in the macrostructure.

### Participants

We tested 44 3- to 5-year-old children exposed to both Italian and Vicentino dialect from birth: 7 3-year-old children (Mean Age: 3.4; Standard Deviation: 2.7 months); 15 4-year-old children (Mean Age: 4.6; Standard Deviation: 4.2 months); 22 5-year-old children (Mean Age: 5.8; Standard Deviation: 3.9 months). In addition, 10 adults (Mean Age: 25.8; Standard Deviation: 6.9 months) were also tested. Children were recruited from three kindergartens in the Vicenza province, located in Thiene, Santorso, and Schio. All participants were typically developing children: none experienced any developmental disorders as attested by the parental report.

Background information concerning the family’s socioeconomic status, length of exposure to Italian and Vicentino, the quantity of the child’s production in both languages, and the quantity of input the child was exposed to in both languages, was collected through the Italian version of the Questionnaire for Parents of Bilingual Children filled out by parents (PABIQ, English version: COST IS0804, 2011; Italian version: see Dicataldo and Roch, 2020; Levorato and Roch, 2020).

According to the parental questionnaire, all children have been exposed to Italian and Vicentino from birth. There were no differences in the socioeconomic status (SES) of the parents: years of education for the mother ranged from 13 to 17 with a mean of 14 years. All children’s parents reported speaking both Italian and Vicentino with their partner at home. Differences emerged in the language they used to interact with their children, the quantity of Vicentino/Italian input their children were exposed to, and, in turn, the language and the quantity of Vicentino/Italian children used in their interactions with the caregivers. Parents were asked to estimate language exposure to both Italian and Vicentino by grading how often they used Italian in comparison to Vicentino when talking with the child and conversely, how often their child replied to the caregiver in either language. Up to five caregivers interacting with the same child were allowed to be listed in the questionnaire. By summing up the grades and dividing the sum for the number of caregivers reported in the questionnaire, we calculated the average amount of time caregivers spoke and children replied in each of the two languages (see Appendix Tables A,B). We identified four ranges for both input and output: only Italian (10), almost exclusively Italian (9.9 > 9), mainly Italian (8.9 > 5,1), and seldom Italian, i.e., mainly dialect (5 > 0). Next, we combined the four ranges of input in Italian with those of output in Italian. Table 1 illustrates the distribution of the 44 children across the Italian input-output ranges.

**TABLE 1:** Children’s distribution according to the quantity of input and output in Italian during their interaction time with caregivers as reported in the Italian version of The Questionnaire for Parents of Bilingual Children (Italian version: Dicataldo and Roch, 2020; Levorato and Roch, 2020).

	Input in Italian			
Output in Italian	100%	99 > 90%	89 > 51%	50 > 0%
100%	13 children	6 children	13 children	1 child
99 > 90%		1 child	4 children	
89 > 51%			3 children	
50 > 0%				3 children

Table 1 shows that most children were exposed to and produce Italian, while only few children received input and replied in Vicentino. Thirteen children received input and replied in Italian only. Moreover, 20 children replied in Italian, although their input was not exclusively in Italian. For the remaining children (*N* = 11), the quantity of Italian in the input seems to match the quantity of Italian in the output. We ran a linear regression to determine whether the quantity of input predicted the quantity of output reported in the questionnaires. Our model revealed a significant effect of the quantity of the Italian input children are exposed to on the quantity of Italian output children use in their interactions (*R*^2^ = 0.0, RMSE = 2.1, *F* = 38.2, *p* ≤ 0.001).

Out of the 44 children, 11 children did not comprehend the questions in the familiarizing phase and the experimental instructions in Vicentino. In the parental questionnaires, these children were reported to use exclusively (*N* = 9) or almost exclusively (*N* = 2) Italian in their interactions with the caregivers. Since we were interested in characterizing bilectal speakers, we excluded these 11 children from further analyses. Hence, the final sample of participants consisted of 33 children: 6 3-year-old children, 9 children aged 4, and 17 5-year-old children.

### Materials and Procedure

Participants were tested on the production and comprehension of Italian and Vicentino dialect with the picture-supported task “Multilingual Assessment Instrument for Narratives” (MAIN, Gagarina et al., 2012, 2015; Gagarina and Bohnacker, 2022), developed within the Narrative and Discourse Working Group (WG2) of the COST Action IS0804, “Language Impairment in a Multilingual Society: Linguistic Patterns and the Road to Assessment.” We adopted the Italian version of the MAIN (Gagarina et al., 2012; Levorato and Roch, 2020). The Italian MAIN was used both in the Telling and the Retelling modes: two picture-supported stories for telling (Baby Birds, Baby Goats) and two for retelling (Cat, Dog), each consisting of three episodes. There were nine comprehension questions for each story, i.e., three questions for each episode. The Italian version of the MAIN was translated into Vicentino. The testing in Italian and Vicentino was carried out by different experimenters and on different days. The testing procedure for narrative elicitation consisted of three stages, the same in each language: (1) Familiarization phase, (2) Narrative Telling (MAIN: Baby Birds/Baby Goats, counterbalanced) and comprehension questions, (3) Narrative Retelling (MAIN: Dog/Cat, counterbalanced) and comprehension questions.

Each child was tested in both Vicentino and Italian throughout four sessions with a delay of at least two days. All children were tested first in Vicentino and then in Italian. Notably, if the story of baby birds was used in Italian in the telling mode, the same child was tested with the other story, i.e., baby goats, in Vicentino. The same observation holds for the Narrative Retelling mode. The instructions, the feedback, as well as the comprehension questions, were given in each language, Vicentino in the Vicentino experiment and Italian in the Italian experiment.

All children were tested individually by a proficient speaker of Italian and Vicentino in a quiet room in the kindergarten. The experimenter and the child were seated next to each other during the telling and retelling modes. First, the child was asked several warm-up questions, e.g., ‘Do you like listening to stories and fairy tales? Do you know what a story or a fairy tale always begins with/ends with?’ If the child did not know the answer, the experimenter explained how stories could begin and end. The child was also prompted to tell any story s/he wanted. Then the experimenter presented the child with three envelopes and informed the child that each contained a different story. Actually, all envelopes contained the same picture story. This step was necessary to strengthen the child’s belief that the experimenter was not familiar with the stories. The child was asked to choose one envelope.

In the telling mode, the child was asked to take the picture story from the envelope, look at the pictures, and tell a story without showing the pictures to the experimenter (the child was explicitly asked not to do that). This was done to ensure the ‘non-shared attention’ condition, as the experimenter was only the listener and the child had to narrate alone. The experimenter prompted the child gently when s/he could not begin, or when there was a long pause. After the production of the story, the experimenter asked the child some questions to assess the child’s understanding of the story. In the retelling mode, when the child had chosen the envelope, the experimenter and the child viewed the pictures together. First, the experimenter told the model story to the child in a friendly manner, following the script and pointing to the pictures (see Gagarina et al., 2012). Subsequently, she asked the child to retell the story. After the retelling, the child was also asked a set of comprehension questions.

### Analysis and Scoring

We analyzed both the macrostructure and the microstructure of the children’s narratives. Macro-structure is the global hierarchical organization of a text and the overall coherence of the story, while microstructure pertains to the specific types of words and syntactic structures that make up the story (Berman and Slobin, 1994). We analyzed four aspects: (a) story structure in both the Telling and the Retelling; (b) comprehension in both modes; (c) syntactic measures; (d) linguistic properties of the production.

#### Story Structure

**FIGURE 1:**
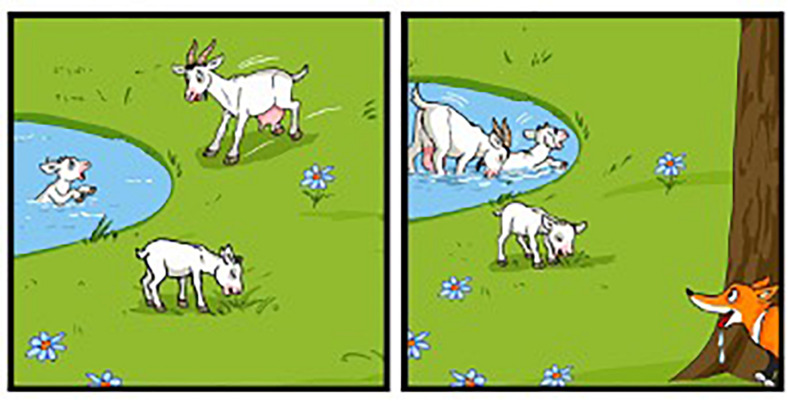
Episode 1 of the Baby goats in the MAIN story telling (from Gagarina et al., 2012).

A maximum of 17 points could be given for story structure in both the Telling and the Retelling mode: 2 points for expressing a setting, and a total of 15 for the three episodes of each story: within each of three episodes 1 point was given for an Initiating event (max. 3 episodes ^∗^ 1 = 3 points); 3 points for each element of the Goal-Attempt-Outcome sequence (max. 3 episodes ^∗^ 3 = 9 points); 1 point for the Reaction/Response (max. 3 episodes ^∗^ 1 = 3 points). We illustrate the scoring with Figure 1, which depicts the first Episode of the Baby Goats story.

The initiating event is represented by the baby goat being scared/in danger/drowning in the water or by the mother seeing the baby goat in danger. The goal is to help the baby and rescue him. The attempt is represented by the mother goat going into the water. The Reaction/Response is the mother goat/baby goat being relieved, happy, not scared anymore.

#### Story Comprehension

A maximum of 9 points could be obtained, 1 for each question answered. All the questions were cued recall questions (see Maviş et al., 2016). Three of the questions targeted the three goals (e.g., ‘Why is the goat in the water?’), two questions elicited internal state terms connected either to the initiating event or reaction elements (e.g., ‘How does the baby goat feel?’), and were followed by three clarification questions (‘Why?’), and one question eliciting a theory of mind response (e.g., ‘Imagine that the bird sees the goats. How does the bird feel?’), followed by a clarification question (‘Why?’).

#### Syntactic Measures

We coded two syntactic measures, namely the number of utterances and the mean length of utterance computed in words. Both measures were calculated independent of the language children used in their narratives. Hence, even if their utterances in the Vicentino mode were in Italian, they were included in the calculation of the number of utterances and the MLU for the Vicentino mode. This choice allowed us to detect whether the language of the instructions in both tasks and of the told story in the retelling task had an effect on children’s performance, by for instance hindering their productions.

#### Linguistic Features

As pointed out by Kupisch and Klaschik (2017), it is not straightforward to determine whether a production qualifies as dialectal or Italian. There is indeed a high degree of linguistic overlap between Vicentino and Italian which regards the lexical, phonological, morphological, and syntactic levels: many words, morphemes, sounds, and structures are identical in the two languages. In addition, as in many bilectal communities, there are mixed codes, intermediate between Italian and the dialect, in the Veneto area. To detect the amount of Vicentino children produce we only considered those phonological, morphological, and syntactic patterns listed in Section “Previous studies on early bilectal acquisition,” where the dialectal grammar diverges from Italian. Therefore, productions were classified as dialectal when they contained a feature that unambiguously qualified as Vicentino according to the dialectological studies. They were classified as Italian when they contained a feature that clearly pertained to Italian grammar. Those elements that could be classified as both Italian and Vicentino, such as the word *luganeghe* ‘sausages,’ were disregarded. We included phonetic properties, such as nasalization, when the audio quality allowed us to analyze children’s pronunciation. Although this procedure allowed us to determine how much dialectal elements were produced, a proper quantitative analysis of children’s dialectal output remains problematic since the data includes a mix of different language levels.

## Results

### Story Structure

We first report results on Story Structure. Table 2 shows the overall scores of Story Structure where children could score a maximum of 17 points in the two narrative modes of the MAIN across the two language modes.

**TABLE 2:** Descriptive statistics for Story Structure in bilingual MAIN-narratives across Age groups: mean and (standard deviation).

	Age	Italian		Vicentino	
		MAIN-Retelling	MAIN-Telling	MAIN-Retelling	MAIN-Telling
Story Structure	3	5.7 (1.2)	3.5 (0.8)	4.3 (1.3)	3.7 (0.8)
	4	6.2 (1.4)	3.9 (1)	4.7 (1.8)	3.64 (1.5)
	5	6.9 (1.9)	5.3 (1.9)	6.9 (1.7)	5.4 (1.5)

Data were fitted to a linear mixed model. The score children obtained in the story structure was our dependent variable. Age (3, 4, 5), Mode (Telling vs. Retelling), Language (Vicentino vs. Italian), and Quantity of Input were the fixed effect factors. Children’s IDs were included as the random effects grouping factor. As for reference categories, ‘3-year-old’ was the reference level for the Age factor, Retelling for the mode factor, and Vicentino for the language factor, and 2 for the Quantity of Input. The model revealed a significant effect of the factor Mode with the retold stories showing a higher scoring for the story structure than the told stories. In addition, a significant effect of Age was detected. No significant effect of Language and Quantity of Input was found and, likewise, no significant interaction of the three factors emerged. The results of the model are reported in Table 3.

**TABLE 3:** Results of the linear mixed model for the dependent variable Story Structure.

Predictors	df	*F*	*p*
Narrative-Mode	1, 27.15	33.95	<0.001
Language-Mode	1, 18.00	1.067	0.315
Age	2, 17.86	6.705	0.007
Quantity of Input	6, 17.86	1.069	0.417
Narrative-Mode * Language-Mode	1, 35.81	4.078	0.061
Narrative-Mode * Age	2, 27.15	0.619	0.546
Language-Mode * Age	2, 18.00	0.993	0.390
Narrative-Mode * Quantity of Input	6, 27.15	0.940	0.483
Language-Mode * Quantity of Input	6, 18.00	0.788	0.591
Age * Quantity of Input	6, 17.86	0.928	0.499
Narrative-Mode * Language-Mode * Age	2, 35.81	0.438	0.649
Narrative-Mode * Language-Mode * Quantity of Input	6, 35.81	1.017	0.430
Narrative-Mode * Age * Quantity of Input	6, 27.15	1.085	0.396
Language-Mode * Age * Quantity of Input	6, 18.00	0.209	0.969
Narrative-Mode * Language-Mode * Age * Quantity of Input	6, 35.81	0.321	0.922

*The model was fitted using restricted maximum likelihood. Full model summary: N = 132, REML 432.2.*

We ran multiple comparisons with Tukey correction on the factor Age. The comparisons revealed that 3-year-old children did not differ from 4-year-old children (MD = –0.3; *SE* = 0.446; *t* = –0.72; *p_*tukey*_* = 0.89) but they differed significantly from the 5-year-old ones (MD = –1.7; *SE* = 0.43; *t* = –3.984; *p_*tukey*_* < 0.001). In addition, 4-year-old children significantly differed from 5-year-old ones. The analyses showed that older children, at age 5, outperformed the younger ones, both 3- and 4-year-olds.

### Story Comprehension

Next, we report results on Story Comprehension. Table 4 illustrates the overall scores of Story Comprehension where children could score a maximum of 9 points in the two narrative modes of the MAIN across the two language modes.

**TABLE 4:** Descriptive statistics for Story Comprehension in bilingual MAIN-narratives across Age groups: mean and (standard deviation).

	Age	Italian		Vicentino	
		MAIN-Retelling	MAIN-Telling	MAIN-Retelling	MAIN-Telling
Comprehension	3	5.8 (1.2)	3.5 (0.9)	4.5 (1)	3.7 (0.8)
	4	6.1 (1.1)	3.9 (1)	4.6 (1.6)	3.7 (1.6)
	5	6.9 (1.8)	5.2 (1.8)	6.9 (1.6)	5.4 (1.5)

We fitted our data to a linear mixed model with one random intercept - participants-, one dependent variable -the score children obtained in the comprehension questions-, and three fixed effects: Age, Mode, and Language. We used the same reference levels outlined in the previous section. The model detected a significant effect of Age and Mode. No significant effect of Language and interactions were found. The results of the model are reported in Table 5.

**TABLE 5:** Results of the linear mixed model for the dependent variable Story Comprehension.

Predictors	df	*F*	*p*
Narrative-Mode	1, 26.61	39.282	<0.001
Language-Mode	1, 18.52	1.042	0.320
Age	2, 18.03	7.283	0.005
Quantity of Input	6, 18.03	1.016	0.446
Narrative-Mode * Language-Mode	1, 36.00	5.330	0.067
Narrative-Mode * Age	2, 26.61	0.465	0.633
Language-Mode * Age	2, 18.52	1.022	0.379
Narrative-Mode * Quantity of Input	6, 26.61	0.946	0.479
Language-Mode * Quantity of Input	6, 18.52	0.748	0.618
Age * Quantity of Input	6, 18.03	0.818	0.570
Narrative-Mode * Language-Mode * Age	2, 36.00	0.533	0.591
Narrative-Mode * Language-Mode * Quantity of Input	6, 36.00	1.378	0.250
Narrative-Mode * Age * Quantity of Input	6, 26.61	1.057	0.412
Language-Mode * Age * Quantity of Input	6, 18.52	0.236	0.959
Narrative-Mode * Language-Mode * Age * Quantity of Input	6, 36.00	0.485	0.815

*The model was fitted using restricted maximum likelihood. Full model summary: N = 132, REML 422.6.*

Tukey *post hoc* comparisons of the main effect of Age showed that 3- and 4-year-old children significantly differed from 5-year-old peers (Age 3 vs. Age 5: MD = –1.6; *SE* = 0.41; *t* = –4; *p_*tukey*_* < 0.001; Age 4 vs. Age 5: MD = –1.4; *SE* = 0.34; *t* = –4.2; *p_*tukey*_* < 0.001). Conversely, 3- and 4-year-old children did not differ (MD = –0.2; *SE* = 0.43; *t* = –0.49; *p_*tukey*_* = 0.96).

### Syntactic Measures

Now we report results on the Syntactic Measures, i.e., number of utterances and mean length of utterance. Table 6 shows the overall scores of Syntactic Measures which children obtained in the two narrative modes of the MAIN across the two language modes.

**TABLE 6:** Descriptive statistics for Syntactic Measures in bilingual MAIN-narratives across Age groups: mean and (standard deviation).

		Age	Italian		Vicentino	
			MAIN-Retelling	MAIN-Telling	MAIN-Retelling	MAIN-Telling
Syntax	N utterances	3	13.2 (6.2)	8.2 (5)	11 (6.4)	6.5 (1.6)
		4	13.3 (4)	8.6 (1.9)	12.5 (4.4)	10.9 (6.4)
		5	14.2 (2.9)	10.9 (3.9)	14.4 (7)	10.9 (4.5)
	MLU (words)	3	6.6 (0.9)	5.6 (0.9)	5.9 (0.8)	5.2 (1)
		4	6.9 (1.8)	5.3 (0.8)	6.2 (1)	5.8 (1)
		5	6.5 (0.9)	6.3 (1)	6.3 (0.8)	5.9 (0.6)

We first analyzed the number of utterances children produced in the two tasks in both Italian and Dialect. We performed the same analyses described for Story Structure and Comprehension. The linear mixed model revealed a significant effect of the factor Mode, with the retelling mode showing more utterances than the telling one. No effects of the other factors and interactions were detected. The results of the model are reported in Table 7.

**TABLE 7:** Results of the linear mixed model for the dependent variable Number of Utterances.

Predictors	df	*F*	*p*
Narrative-Mode	1, 18.03	12.439	0.002
Language-Mode	2, 18.11	1.375	0.278
Age	1, 18.57	0.861	0.365
Quantity of Input	6, 18.11	1.478	0.241
Narrative-Mode * Language-Mode	2, 18.03	0.317	0.733
Narrative-Mode * Age	2, 18.57	0.508	0.610
Language-Mode * Age	1, 36.00	0.108	0.744
Narrative-Mode * Quantity of Input	6, 18.11	2.042	0.112
Language-Mode * Quantity of Input	6, 18.03	0.352	0.900
Age * Quantity of Input	6, 18.57	0.658	0.684
Narrative-Mode * Language-Mode * Age	2, 36.00	1.086	0.348
Narrative-Mode * Language-Mode * Quantity of Input	6, 18.03	0.677	0.670
Narrative-Mode * Age * Quantity of Input	6, 18.57	1.381	0.273
Language-Mode * Age * Quantity of Input	6, 36.00	2.230	0.062
Narrative-Mode * Language-Mode * Age * Quantity of Input	6, 36.00	1.257	0.302

*The model was fitted using restricted maximum likelihood. Full model summary: N = 132, REML 568.8.*

Next, we analyzed the mean length of children’s utterances by calculating the number of words. As for the number of utterances, the model revealed a significant effect of only the Mode factor, with the retold stories containing a higher number of words than those produced in the Telling mode. The results of the model are reported in Table 8.

**TABLE 8:** Results of the linear mixed model for the dependent variable MLU.

Predictors	df	*F*	*p*
Narrative-Mode	1, 21.58	32.726	<0.001
Language-Mode	1, 18.02	0.858	0.367
Age	2, 18.04	1.451	0.260
Quantity of Input	6, 18.04	0.763	0.608
Narrative-Mode * Language-Mode	1, 36.00	0.069	0.795
Narrative-Mode * Age	2, 21.58	1.526	0.240
Language-Mode * Age	2, 18.02	0.312	0.736
Narrative-Mode * Quantity of Input	6, 21.58	2.323	0.070
Language-Mode * Quantity of Input	6, 18.02	2.067	0.109
Age * Quantity of Input	6, 18.04	0.373	0.887
Narrative-Mode * Language-Mode * Age	2, 36.00	4.549	0.067
Narrative-Mode * Language-Mode * Quantity of Input	6, 36.00	2.150	0.071
Narrative-Mode * Age * Quantity of Input	6, 21.58	1.668	0.177
Language-Mode * Age * Quantity of Input	6, 18.02	0.440	0.842
Narrative-Mode * Language-Mode * Age * Quantity of Input	6, 36.00	0.659	0.683

*The model was fitted using restricted maximum likelihood. Full model summary: N = 132, REML 355.*

Taking the results together, the statistical analyses revealed a main effect of the Mode factor. The stories produced in the Retelling task showed higher scores in the Story Structure and the Comprehension questions and elicited a higher number of utterances with a higher mean length of utterance than the stories produced in the Telling task. In addition, Age had an effect on the Story Structure and the Comprehension questions but not on the Syntactic Measures: 5-year-old children obtained better scores than their 3- and 4-year-old peers. Quantity of Input did not yield any significant effect. Importantly, no effect was detected in the language of the experiment, whether Italian or Vicentino.

However, it should be stressed that, when looking at their production in the two versions of the tasks, we noticed that the 33 children completing both versions never produced an entire story in dialect. Despite understanding the comprehension questions and the instructions in Vicentino, their replies and their narratives were mainly in Italian. Out of 2440 words produced in the Vicentino Retelling task, we counted 86 dialectal words, 1722 words in Italian, and 632 words which we could not determine as Italian or Vicentino. Out of 2037 words produced in the Vicentino Telling task, we counted 41 words that we could classify as dialectal and 1523 words in Italian. We could not assign a value to 473 words. In the Italian experiments, the presence of dialectal features was even lower. Out of 2607 words in the Italian Retelling task, 4 words were Vicentino, while 1832 were clearly Italian. In the Italian Telling task, 8 out of 2073 words were dialectal and 1788 were Italian.

This shows that there is a discrepancy between comprehension and production of the dialect. While all 33 children comprehended the dialect, not all of them produced Vicentino.

### Linguistic Features

Next, we analyzed children’s productions from a qualitative viewpoint to detect the amount and type of dialectal elements present in the stories. On the basis of the dialectal elements produced by the 33 children, we delineated three profiles.

The first profile comprises five children who comprehended the instructions and the questions in Vicentino but did not produce any dialectal element at the phonological, lexical, morphological, or syntactic level. Interestingly, in the parental questionnaires, these 5 children were reported to exclusively use Italian in their interactions with the caregivers. In addition, the quantity of dialectal input these children were reported to receive was either null or very scarce (see Appendix Tables A,B).

The remaining 28 children produced a story mainly in Italian but with some dialectal interference present. All 28 children produced some dialectal elements in the Vicentino experiment and did so more in the Retelling story (*N* = 106) than in the Telling story (*N* = 41). Three children also produced 12 dialectal elements in the Italian Telling and Retelling tasks: these were mainly lexical elements like *toso*, *cana*, *bala*, but in four occurrences children produced the dialectal auxiliary selection, as in *se l’ha ripreso* ‘he took it back.’

Among the 28 children who produced some Vicentino elements, 19 children comprehended the instructions and the questions and produced phonological, morphological, and lexical dialectal elements. This represents the second profile. All of them were reported to exclusively or mainly use Italian in their interactions with the caregivers (see Appendix Table A). Eleven children were exposed to some dialect for at least 12% of the interaction time with the caregiver, while the remaining eight children were reported to receive very little or null dialectal input (see Appendix Table B).

Among the phonological dialectal features, all these children produced apocope of the final vowels/e/and/o/following/n/as illustrated in (4), with the consequent velarization of the nasal consonant. Out of 560 produced words that contained potential contexts for apocope to apply, 84 words were produced with apocope. In 38 cases we were able to determine that children also produced the final nasal consonant as a velar nasal.


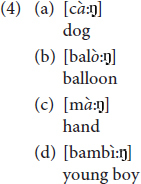


As noticed in the dialectological literature, in Vicentino/e/and/o/occurring in word-final position do not undergo apocope when they are preceded by other consonants, and neither do/r/nor/l/, unlike the Venetian dialect. Interestingly, all children respected this rule as shown by the productions in (5) where /e,o/ follow consonants different from/n/and are not dropped.


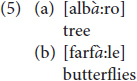


In intervocalic context/t, l/ are degeminated (6a,b) and voiceless plosives become voiced as in (6c,d). For those items where degemination could have occurred, children always produced it. Children never produced the dialectal words without degemination, e.g., *balla* instead of *bala*. Children either produced it with degemination or they produced the Italian lexeme *palla*, without degemination.


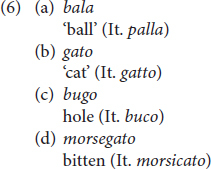


As for the morphological dialectal features, all children produced the Vicentino weak form of the masculine singular article/el/: *el can* ‘the dog,’ *el balon* ‘the balloon,’ *el toso* ‘the young boy.’ Out of a total of 422 definite articles, 286 were Vicentino, while the remaining articles were Italian. Sometimes, the Vicentino article also introduced Italian nouns (*N* = 45), as in *el cane* ‘the dog.’

Some children also produced predicates with clear dialectal prefixes. One case is represented by the verb *incorzarse* ‘to realize’ with the prefix *in-* while the corresponding Italian predicate has the prefix *a*-, *accorgersi*: *el can se incorze che* […] ‘the dog realizes that […]’. Another case is the verb *scumissiare* ‘to begin, start’ with the prefix *s-* instead of the corresponding Italian *in*-, *incominciare*.

The dialect was also present in other lexical items. For instance, all 19 children used the verb *ciapare* ‘to take, catch’ at least once: *el can voe ciapare el topo* ‘the dog wants to catch the mouse.’ In a few cases, some children also used the verb in the idiomatic use with the noun *paura* ‘scare,’ as in *el toso ciapa paura* ‘the boy got scared.’

Finally, the third profile comprises nine children who comprehended the instructions and the questions in dialect and, in addition to phonological and lexical dialectal elements, also produced syntactic dialectal structures. Overall, the profile matched the expectations we had, based on the parental questionnaire. This especially holds for three children who were reported to mainly receive dialectal input and in turn to reply in Vicentino. Likewise, two children were reported to receive input and reply in Vicentino for 33% of their interaction time with the caregivers. However, for four children our analysis diverged from the results of the parental questionnaire: they were reported to be exposed to dialect for at least 33% of their child–caregiver interaction time but to use Italian either exclusively or almost exclusively (see Appendix Table B). In addition to the phenomena quoted for the second profile, these nine children produced dialectal syntactic structures, all of which occurred in code-switched utterances. Interestingly, the dialectal syntactic structures produced by the nine children share one commonality: they are all related to the lower portion of the clause, involving aspectual, voice, and tense functional projections. Conversely, subject clitics and the locative/dative clitic *ghe*, which are clear syntactic properties of Vicentino, were not present in any of the children’s productions. Likewise, complementizers were all produced in Italian: *quando* ‘when’ instead of the dialect *cò, perché* ‘because’ instead of *parché, come* ‘as’ instead of *come che*.

In at least one instance, all children produced particle verbs. Some examples are *tornare zò* ‘lit. to go back down; to climb down,’ *saltare drio* ‘lit. to jump behind; to chase,’ *corere drio* ‘lit. to run behind; to chase,’ *abaiare drio* ‘lit. to bark behind; to bark,’ *molare zò* ‘lit. to release down; to release.’ The particle verb was also produced when the phonological shape of the lexemes was Italian, as in the case of *va su per l’albero* ‘he climbs the tree.’

Another domain in which dialectal production was detected was the selection of the auxiliary. With reflexive, impersonal, and modal verbs, Vicentino selects the auxiliary ‘to be,’ while Italian has the auxiliary ‘to have.’ Children produced the structures in (7) with the auxiliary verb ‘to be.’


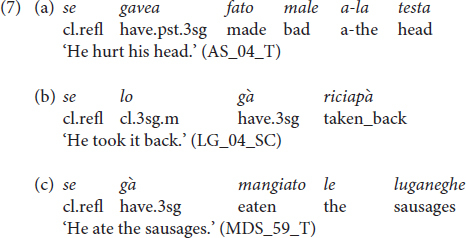


Finally, some children also produced the imperfective progressive construction typical of the Vicentino dialect and ungrammatical in Italian. The periphrasis is formed by the auxiliary *essere* ‘to be,’ the adverb *drio* ‘behind’ and a bare infinitive. This is illustrated in (8). Notice that in (8a), the structure is dialectal as well as the adverb *drio*, but the complementizer, the null subject, and the lexical predicate are Italian. Likewise, in (8b), the periphrasis is dialectal: the adverb and the lexical predicate are Vicentino, but the null subject and the form of the direct object matches with Italian.


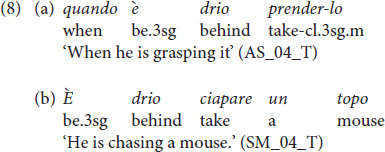


## Discussion

This paper investigated children’s linguistic competence in a standard language, namely Italian, and a vernacular non-standardized local dialect, namely the Vicentino dialect. Forty-four children from age 3–5 were tested with two narrative tasks in both Italian and Vicentino. We asked four questions: (Q1) Does children’s performance in narrative comprehension and production differ depending on the mode of the narrative task, i.e., retelling vs. telling?; (Q2) Are there differences in narrative abilities between children determined by age?; (Q3) Does children’s performance in narrative comprehension and production differ depending on the language of the experiment, i.e., Vicentino vs. Italian?; (Q4) Does children’s competence depend on the quantity of input?.

### (Q1) The Role of Narrative Mode

We found a significant effect of the narrative mode on both the macrostructure and the microstructure measures, showing that retelling elicited more coherent, articulated and longer stories than telling. In addition, the retelling task enhanced the comprehension of the story. Our results met our expectations and are in line with previous findings on bilinguals. Various studies on bilinguals found that bilingual children comprehended better and produced more structured and coherent stories with the Retelling than with the Telling task (e.g., Kunnari et al., 2016; Maviş et al., 2016; Roch et al., 2016; Bonifacci et al., 2018; Levorato and Roch, 2020; Roch and Hržica, 2021). While the literature generally agrees that the Retelling task enhances the macrostructure, the results on the role of the narrative mode for the microstructure are more controversial. Some studies found that presenting the child with a model story improved the lexical and syntactic complexity of the story (e.g., Adlof et al., 2014). Conversely, others reported that the microstructure improved in the Telling task (e.g., Gutiérrez-Clellen, 2002). In our case, the retold stories contained more utterances and exhibited a longer MLU. Hence, Retelling seems to positively affect these two syntactic measures. This observation also holds for the number of dialectal elements elicited, which was higher in the Retelling task than in the Telling.

### (Q2) The Role of Age

We found a main effect of age in the macrostructure, showing that the complexity and accuracy of children’s narratives grow with children’s ages. Three- and four-year-old children differed from their older peers in both story structure and comprehension. Our findings are in line with previous studies which demonstrated that children’s narrative abilities increase with age (e.g., Pearson, 2002; Florit et al., 2014; Bohnacker, 2016; Bonifacci et al., 2018).

### (Q3–4) The Role of Language-Mode and Input

The language of the experiment, Italian vs. Vicentino, did not yield any significant effect in children’s responses in the macrostructure and, to some extent, in the microstructure measures as well. This result corroborates earlier findings on bilinguals, where macrostructure measures also remained relatively invariant across bilingual children’s languages (Pearson, 2002; Fiestas and Peña, 2004; Gagarina et al., 2015; Kunnari et al., 2016). Story macrostructure has usually been claimed to be less dependent on language abilities as compared to microstructure (Berman and Slobin, 1994; Rodina, 2016). We also found no effect of language in the two microstructure measures, i.e., the number of utterances and the MLU. Notice, however, that when we looked into children’s production, the majority of lexemes and structures in both experiments were Italian. As clarified in Section “Results,” children responded mostly in Italian in the Venetan mode. The overabundance of Italian in the Vicentino experiment may be the reason why we did not find substantial differences in the microstructure measures across the two experiments. If the children had responded in Venetan, there might have been differences. Yet, it is interesting to note that, even if children’s productions mainly consisted of an Italian lexicon and morphosyntax, the use of Vicentino in the instruction, the model story, and the comprehension questions did not negatively affect children’s production. A clear difference due to the language-mode emerged in the linguistic feature analysis, to which we will return later.

As seen for language, quantity of input was also not detected as a significant factor in the macrostructure. Our result is in line with previous findings by Gagarina (2016) on Russian–German bilinguals, Bohnacker (2016) on Swedish–English bilinguals, Kunnari et al. (2016) on Finnish–Swedish children, Iluz-Cohen and Walters (2012) on English–Hebrew, and Rodina (2016) on Russian–Norwegian bilinguals. These studies found that narrative macrostructure is relatively invariant across languages and is not much reliant on language proficiency, while narrative microstructure seems to be more dependent on language proficiency. However, we also find no effect of language exposure in the microstructure measures. As for the language-mode factor, this finding may be explained by children’s overuse of Italian in the Vicentino experiment. Indeed, children’s productions mainly consisted of Italian lexical items and syntactic structures in both experiments. Conversely, the results on the parental questionnaire showed that the quantity of dialectal input children are exposed to positively correlates with the quantity of dialect children were reported to use. Likewise, the linguistic profiles drawn from the parental questionnaires generally matched the results on the linguistic feature analysis, suggesting that language exposure does play a role in the production of dialect, as suggested in previous studies on Venetan-Italian children (Klaschik and Kupisch, 2016; Kupisch and Klaschik, 2017).

Our study further showed that all the 44 preschool children that were reported to have been exposed to dialect and Italian from birth produced and comprehended Italian. Out of them, 11 children were classified as monolingual Italian speakers since they failed to understand the questions in the familiarization phase or the experimental instructions in Vicentino. Interestingly, in the parental questionnaire, these children were reported to use Italian exclusively or almost exclusively in their interactions with the caregivers. Among them, 10/11 children were reported to receive scarce or null input in the Vicentino dialect in the interactions with their caregivers. We found one exception: one child did not understand the dialect questions although she was reported to receive dialectal input 43% of the time. Overall, we can conclude that quantity of input plays a role in the comprehension of the dialect.

The remaining 33 children comprehended Vicentino. Depending on the quantity and type of Vicentino elements in their productions, we identified three linguistic profiles. The first profile is represented by receptive bilinguals (Mioni, 1979), comprising five children who comprehended the instructions and the questions in Vicentino but did not produce any dialectal element at the phonological, lexical, morphological, or syntactic level. Interestingly, in the parental questionnaire, these five children were reported to exclusively use Italian in their interactions with the caregivers and to receive either null or very scarce quantity of dialectal input. For these children as well, the quantity of exposure seems to play a role in the production of dialect. The second profile is represented by 19 bilinguals who mainly produced their stories and replied in Italian but with phonological and morphological dialectal elements. For this group of children, we did not find any stable link with the quantity of input reported in the parental questionnaire: all children were reported to exclusively or mainly use Italian in their interactions with the caregivers; 11 children were exposed to some dialect for at least 12% of the interaction time with the caregiver; 8 children were reported to receive very little or null dialectal input. The third profile is represented by nine children who produced some dialectal elements at the phonological and morphological level, as in the second profile, but also at the syntactic level. Overall, the children in this profile matched the expectations we had, based on the results from the parental questionnaire. All children were reported to mainly receive dialectal input or in at least 33% of their interaction time with the caregivers (see Appendix Table B).

We may conclude that, with some exceptions, the quantity of Vicentino input children receive has a positive effect on children’s comprehension and production abilities of the minority language. Our results nicely match those on Venetan in Kupisch and Klaschik (2017). Infrequent dialect users were found to rely more on Italian than frequent dialect users. The authors suggested that children’s dialectal production depends on the quantity and quality of the dialectal input. Our data nicely confirm this conclusion. Conversely, our results diverge from the findings on Friulian in Covazzi (2019): the author did not find a correlation between children’s Friulian production and the quantity of Friulian input reported in the parental questionnaires. Various reasons, both methodological and linguistic, may account for this difference. One factor may be the different sizes of the participants’ samples: while we tested 44 children, Covazzi analyzed the production of 23 children aged 4-to-6 years. Given the high degree of variation we and Covazzi as well found in the parental responses, a smaller sample may not be sufficient to draw any generalization. Another reason may lie in the scale used to evaluate the quantity of input: while our scale had 10 points, Covazzi adopted a scale with four levels, i.e., ‘almost always,’ ‘often,’ ‘sometimes,’ ‘almost never.’ Participants may have faced difficulties in quantifying the dialectal input with a non-numerical scale. Finally, the differences between our and Covazzi’s results may reflect the different sociolinguistic profiles of Friulian and Venetan (Vanelli, 2005; Vicario, 2015).

The linguistic analysis also suggests an implicational scale in the dialectal competence: if a child exhibits some productions with dialectal syntax, s/he also produces dialect at the phonological, morphological, and lexical level, but not vice versa. From a theoretical perspective, it is interesting to note that the dialectal syntactic structures children produced are all related to the lower portion of the clause, involving verbal, voice, and aspectual functional projections. On the other hand, the higher layers of the clause, where subject and locative clitics, as well as complementizers, are merged, are only Italian. This result is consistent with the Growing Tree Hypothesis proposed by Friedmann et al. (2020). According to this view, children’s developmental stages follow the geometry of the syntactic tree (see Rizzi, 2004): early stages correspond to small portions of the adult syntactic tree, which grows during development. Although further studies are in order, it seems plausible to extend this approach to bilectal acquisition as well.

## Conclusion

Our results demonstrated that dialectal competence is already present in preschool children. In addition, our findings suggest that dialectal competence should be arranged along a fine-grained continuum. Unlike “standard” bilingual speakers, bilectal speakers can comprehend dialect although they may completely lack the competence to produce it. In this sense, they can be qualified as receptive bilinguals. Bilectal speakers may also produce some dialectal elements. Some bilectal children only have access to the phonological, morphological, and lexical domain of the dialect, while others also produce dialectal syntactic structures. Although the results on the macrostructure are similar to the findings from standard bilingual studies, the strikingly small number of dialectal elements produced by children suggests that bilectal acquisition may be different from standard bilingualism. This may be a reflection of the sociolinguistic differences outlined in Section “Introduction,” but also of the linguistic challenges discussed in Section “Analysis and scoring.” As reported in previous studies on bilectal acquisition, there is indeed a high degree of linguistic overlap between Vicentino and Italian which regards the lexical, phonological, morphological, and syntactic levels. As a result, it is not straightforward to determine whether a production qualifies as dialectal or Italian and in turn to provide an appropriate measure of how much dialect children produce. For future work, it would be relevant to test bilectal children with different experimental methods, such as grammaticality judgment tasks, to tease apart the lack of production of a given structure from the lack of grammatical competence of that structure. Moreover, since the dialectological profiles in Italy differ from one region to another, future studies on different dialects are necessary to establish the role of the extra-linguistic factors on bilectal acquisition. As a matter of fact, our results may be extended to other regions with a context similar to Veneto, maybe Apulia and Basilicata, but not necessarily to others, like Liguria or Lombardy.

## Data Availability Statement

The original contributions presented in the study are included in the article/supplementary material, further inquiries can be directed to the corresponding author/s.

## Author Contributions

All authors listed have made a substantial, direct and intellectual contribution to the work, and approved it for publication.

## Conflict of Interest

The authors declare that the research was conducted in the absence of any commercial or financial relationships that could be construed as a potential conflict of interest.

## Publisher’s Note

All claims expressed in this article are solely those of the authors and do not necessarily represent those of their affiliated organizations, or those of the publisher, the editors and the reviewers. Any product that may be evaluated in this article, or claim that may be made by its manufacturer, is not guaranteed or endorsed by the publisher.

## Footnotes

1 The remaining percentage refers to the use of another (foreign) language.

2 Abbreviations in examples follow Leipzig’s glossing conventions.

## Appendix

**TABLE A:** Overview of the linguistic profiles of the children according to children’s input and output in Italian reported in the Italian version of The Questionnaire for Parents of Bilingual Children (Italian version: Dicataldo and Roch, 2020; Levorato and Roch, 2020).

Italian input	Italian output	N of children
10	10	13
9.6	10	2
9.5	9.5	1
9.5	10	1
9.2	10	2
9	10	1
8.8	10	3
8.6	9.2	1
8.5	10	1
8.4	10	2
8.3	10	1
8	10	3
7.2	10	1
7.2	8.5	1
7	10	1
6.8	9	1
6.7	6.7	1
6.7	10	1
6.6	8.4	1
6.5	9	1
5.7	9	1
4.3	1	1
3	1.5	1
2.3	2	1
1.5	10	1

**TABLE B:** Overview of the linguistic profiles of the children according to children’s input and output in dialect reported in the Italian version of The Questionnaire for Parents of Bilingual Children (Italian version: Dicataldo and Roch, 2020; Levorato and Roch, 2020).

Dialectal input	Dialectal output	N of children
0	0	13
0.4	0	2
0.5	0.5	1
0.5	0	1
0.8	0	2
1	0	1
1.2	0	3
1.4	0.8	1
1.5	0	1
1.6	0	2
1.7	0	1
2	0	3
2.8	0	1
2.8	1.5	1
3	0	1
3.2	1	1
3.3	3.3	1
3.3	0	1
3.4	1.6	1
3.5	1	1
4.3	1	1
5.7	9	1
7	8.5	1
7.7	8	1
8.5	9	1
